# Exploring the Early-Life Microbiomes of Preterm Infants and Their Childhood Health Outcomes (The BLOOM Study): Protocol for a Prospective, Observational Cohort Study

**DOI:** 10.2196/95658

**Published:** 2026-08-18

**Authors:** Michelle R Asbury, Andrea Guedez, Jessica Lee, Emily M Mercer, Fatimoh Kasaba, Zahra Rangipour, James D Kellner, Thierry Lacaze-Masmonteil, Jumana Samara, Lara M Leijser, Catherine Lebel, Hussein Zein, Leonora Hendson, Luis Murguía Favela, Kelly McNagny, Gerald F Giesbrecht, Laura Sycuro, Belal Alshaikh, Marie-Claire Arrieta

**Affiliations:** 1 Department of Physiology & Pharmacology, University of Calgary, 3330 Hospital Dr NW, HRIC Room 4A25, Calgary, AB, T2N 1N4, Canada, 1 403-220-7803, 1 403-220-7803; 2 Department of Pediatrics, Cumming School of Medicine, University of Calgary, Calgary, AB, Canada; 3 Snyder Institute for Chronic Diseases, University of Calgary, Calgary, AB, Canada; 4 Alberta Children’s Hospital Research Institute, Calgary, AB, Canada; 5 Department of Community Health Sciences, Cumming School of Medicine, University of Calgary, Calgary, AB, Canada; 6 Section of Hematology and Immunology, Alberta Children’s Hospital, University of Calgary, Calgary, AB, Canada; 7 Hotchkiss Brain Institute, University of Calgary, Calgary, AB, Canada; 8 Biomedical Research Centre, Faculty of Medicine, University of British Columbia, Vancouver, BC, Canada; 9 Department of Microbiology, Immunology & Infectious Diseases, Cumming School of Medicine, University of Calgary, Calgary, AB, Canada

**Keywords:** prematurity, microbiome, asthma, allergy, immune development, neurodevelopment, cohort study

## Abstract

**Background:**

Establishment of the gut microbiome during the first years of life is critically important for long-term health and development. In preterm infants, microbial colonization is disrupted due to their developmental immaturity and the myriad of pre- and postnatal exposures. Unfortunately, this places them at an elevated risk for adverse health outcomes into childhood (eg, asthma and impaired neurodevelopment). Understanding the developmental trajectory of the microbiome in preterm infants, the factors predicting these microbial patterns, and the links to childhood health can offer opportunities to develop interventions and optimize health.

**Objective:**

BLOOM (Begin a Life of Health With Observation and Optimization of the Microbiome) is a prospective, observational cohort study that aims to examine how early-life microbiomes shape the health and development of preterm infants into childhood (eg, asthma and neurodevelopment).

**Methods:**

Infants (<37 weeks gestation) and their families are recruited from 4 neonatal intensive care units in Calgary, Alberta, Canada. Maternal stool and weekly infant stool, urine, and human milk samples are collected during the first 2 months postnatally; participants are then followed up at 3 months, 1 year, and 3 years corrected age for continued data (eg, nutrition, medications, medical history, and home environment) and sample collection (stool, urine, nasal swabs, hair, and blood). Additional clinical testing (eg, allergen skin prick test) and questionnaires (eg, Ages & Stages Questionnaires, Third Edition) are administered to assess health outcomes and developmental milestones.

**Results:**

Study recruitment and data/sample collection for BLOOM commenced in 2019; as of May 2026, we have enrolled 245 participants, and recruitment is ongoing. An initial characterization of early-life microbiome profiles for the first 105 infants enrolled in BLOOM was published online in December 2025.

**Conclusions:**

BLOOM is a large, comprehensive prospective cohort study and biobank aimed at measuring the microbiome development of preterm-born children. This cohort will address significant research priorities through characterizing the patterns of microbiome development in preterm infants over the first 3 years postnatally and correlating these with health outcomes (eg, immune development, asthma and allergy risks, and neurodevelopment). It is anticipated that the results can be leveraged to identify factors and altered microbiome patterns underlying disease risk, design intervention strategies (eg, microbial therapeutics) for later clinical testing, and generate novel hypotheses of possible underlying mechanisms linking microbiome development to health.

## Introduction

### Microbiome Disruptions in Preterm Infants

There is a growing appreciation for the symbiotic relationship between humans and their microbiome to support healthy development. Early microbial colonizers of the gastrointestinal tract play a pivotal role in the type of microbial community structure that forms during infancy and childhood. However, this process can be interrupted by several pre-, peri-, and postnatal factors that commonly occur in early life, such as cesarean section (C-section) delivery, antibiotic exposure, and formula feeding [1,2]. This is especially true for infants born preterm (<37 weeks gestation) who may experience a number of aberrant prenatal and postnatal exposures, combined with their structurally and functionally immature gut [3-5]. Alterations to the gut microbiota in preterm infants are common and associated with an elevated risk of numerous disorders, including necrotizing enterocolitis, late-onset sepsis, and impaired neurodevelopment [3-7]. However, the specific microbiome features involved in acute and long-term disease development, and the underlying mechanisms that lead to disease progression and poor neurodevelopment, represent one of the least understood frontiers in pediatric care.

### Preterm Infants

Being born preterm is one of the leading causes of pediatric morbidity and mortality worldwide [8,9]. In Canada alone, 8% of infants are born preterm (approximately 29,000 infants every year), with the earliest gestational ages having the highest risks of health complications [10,11]. Prematurity is a spectrum, with the earliest born infants classified as extremely preterm (<28 weeks gestation), followed by very (28‐32 weeks), moderately (32‐34 weeks), and late (34‐37 weeks) preterm. Unsurprisingly, infants born extremely and very preterm are more likely to experience greater clinical interventions than those born at later gestational ages, such as greater antibiotic administration, respiratory support (eg, mechanical ventilation), intravenous and enteral tube feeding, stress-inducing procedures (eg, surgery and frequent blood draws), reduced contact with parents, and extended hospital stays [12]. Furthermore, being born preterm predisposes individuals to developing numerous health complications into childhood and adulthood, including asthma, metabolic syndrome, and impaired neurodevelopment [13-17]. The earlier an infant is born, the greater the risk of these health complications along with poorer rates of employment, lower income, and reduced enrollment in higher education [13-18]. Therefore, interventions aimed at targeting microbiome development and the health of preterm-born children will depend upon their gestational age at birth and associated early-life exposures, with important implications for their overall health and well-being throughout life.

### Microbiome Development

The term “microbiome” encompasses microbial communities, along with their functions (eg, metabolites) in the surrounding environment [19]. Microbiomes reside in numerous body sites including, but not limited to, nasal, oral, vaginal, and skin microbiomes; however, the gut is the most widely studied microbiome to date.

During the first months of life, the patterns of gut microbiome development in preterm infants are altered compared to term-born infants, which becomes more pronounced with earlier gestational ages at birth [20,21]. There are numerous reasons for this, including immaturity of the gastrointestinal and immune systems, higher likelihood of antibiotic exposure pre- and postnatally, greater likelihood of C-section delivery, increased risk of exposure to pathogenic microbes pre- and postbirth, reduced contact with parents, and reduced mother’s milk feeding—all of which are associated with altered early colonization patterns of the infant gut [3-5]. In order to gain a thorough understanding of these altered microbial patterns, a comprehensive evaluation of perinatal factors (eg, maternal microbiome, pregnancy complications, delivery mode, nutrition, and antibiotics) and their effects on the preterm microbiome, metabolome, and immune development over time is needed.

Microbiomes established in early life have the potential to shape the trajectory of microbial development and sculpt the immune system to thereby influence health outcomes into childhood [2,22]. For instance, the nasal microbiome during the first few weeks postnatally has shown links to bronchopulmonary dysplasia risk in preterm infants later in hospitalization [23]. For the gut microbiome, it is thought to stabilize by approximately 3 years of age, offering an early window of opportunity to modify [1,2]. Although the gut microbiome of preterm- versus term-born children generally converges over the first 1‐3 years of life, there are often still microbial and metabolite signals that can differentiate these gestational age groups, which are thought to be driven by pre- and postnatal exposures (eg, delivery mode, human milk feeding, and pharmacological treatments) [24-28]. This is especially true among preterm infants who experience serious morbidity during the neonatal period, such as necrotizing enterocolitis [29]. Furthermore, the early-life gut microbiome in preterm infants has been associated with later health outcomes into childhood. For instance, Rozé et al [30] surveyed 577 very preterm infants across France and found that gut microbiome patterns dominated by *Enterococcus*, *Staphylococcus,* or low bacterial loads at 4 weeks postnatally were associated with a greater risk of suboptimal neurodevelopment (as measured by the Ages & Stages Questionnaires, Second Edition [ASQ-2]), cerebral palsy, and/or death by 2 years corrected age (CA).

### Asthma and Allergies

Asthma is a leading cause of school absenteeism, pediatric hospitalizations, and adult work loss [31-33]. This debilitating disease affects approximately 1 in 9 Canadian children with a total economic burden of CAD $2.20 billion/year in 2010 (CAD $1=US $1.02 as of November 1, 2010)—an amount that is expected to grow to CAD $4.20 billion by 2030 [34,35]. In Canada and globally, preterm infants are at 1.7 times greater risk of developing childhood respiratory disorders, including asthma, with those born extremely or very preterm having a 3 times greater risk than infants born at term, likely due to several mechanisms that are unique to the preterm population [36]. Lung immaturity and immune dysregulation, combined with the need for respiratory support, can induce inflammation and long-term structural lung damage, subsequently leading to bronchopulmonary dysplasia [37]. Preterm infants also experience immune system immaturity, rendering them susceptible to respiratory infections. In fact, experimental animal models that use antibiotics to shift maternal and neonatal microbial communities suggest that, mechanistically, these microbial shifts can have a profound effect on the stereotypic development of lymphocyte and innate immune cell subsets, with long-term consequences in adult responses and susceptibility to disease [22]. Lastly, exposure to stress (eg, medical procedures and enhanced exposure to sensory stimuli in the neonatal intensive care unit) can alter microbiota-to-brain signaling via dysregulation of the hypothalamic-pituitary-adrenal stress axis. Together, their immature immune system and external stress exposures can alter asthma susceptibility [38-41]. Recent studies have provided evidence of a causal link between early-life gut microbiome alterations and the development of asthma; however, these have largely excluded preterm infants, despite them being at the highest risk of developing asthma [42-46].

Although preterm infants are at an elevated risk of immune dysregulation and asthma, there is little data exploring their risk of developing a food or environmental allergy. Findings from observational cohort studies have been mixed, although generally showing a lower risk of IgE sensitization with earlier gestational ages [47-50]. However, from a microbiome perspective, recent findings from the Canadian Health Infant Longitudinal Development (CHILD) cohort (>1000 children across Canada) have shown that delays in microbiome development at 1 year strongly predict allergy diagnoses by the age of 5 in term-born children [51]. Given the unique postnatal exposures and altered patterns of microbial colonization observed among preterm infants, the crosstalk between microbial species, their metabolic byproducts, and the mechanisms relevant to asthma and allergy development in preterm infants may differ from those born at term.

### Neurodevelopment

Preterm birth is a significant determinant for impaired neurodevelopment in infancy and into adulthood [52]. Similar to asthma, infants born preterm are significantly more likely to develop neurodevelopmental issues than their term-born counterparts, including cerebral palsy, developmental disabilities (eg, intellectual/cognitive delays, speech and language disorders, and neuro-motor disorders), attention-deficit/hyperactivity disorder, autism spectrum disorder, and psychiatric disorders in adulthood [53-60]. Head circumference growth is often used as a proxy for brain development, with some evidence showing slower growth being linked to lower total cerebral volumes and poorer neurodevelopment in childhood [61,62]. Although impaired neurodevelopment in preterm infants is multifactorial, the gut microbiome and its actions via the gut-brain axis are thought to play an important role. In a recent narrative review by Beghetti et al [7], specific microbial signatures (eg, pathogenic microbes and lower *Bifidobacterium* abundance) were linked to head circumference growth, performance on developmental scales, and clinical measures (eg, electroencephalogram) across different preterm infant cohorts in early life and into childhood. This is likely due to mechanisms including microbially derived metabolites and hormones, and the induction of proinflammatory cytokines (particularly from pathogenic microbes and infections), which can all mediate brain maturation [7].

### Study Objectives

The overarching goals of this cohort are to comprehensively study microbiome development in preterm infants and understand how microbial patterns during this critical early-life window contribute to development and disease risk in childhood. The primary objective for this cohort is to profile microbiome development from birth to 3.5 years CA in children born preterm (≤36^+6/7^ days gestation) and compare it to children born at term.

The secondary objectives are (1) to determine how perinatal factors are associated with microbiome development in children born preterm versus at term and (2) to determine the links between microbiome development and infant outcomes at 1- and 3-years CA, including the Asthma Predictive Index and other atopy such as food and environmental allergies.

Exploratory objectives are (1) to examine the role of environmental, nutritional, and pharmacological exposures in the development of the preterm and term infant microbiome, metabolome, and immunobiome; (2) to explore correlations between the gut microbiome, early-life stress, and fronto-amygdalar brain connectivity at 1 month CA; and (3) to establish how microbiome development in preterm infants is associated with head circumference at term CA and with neurodevelopment at ages 1 to 5.5 years.

We hypothesize that specific microbial patterns and/or metabolites will be significantly associated with prenatal, maternal, and/or infant factors (or combinations of factors). As well, we hypothesize that microbial alterations resulting from preterm birth causally contribute to the higher rates of allergies and asthma observed in preterm infants through immune mechanisms, which differ from those in infants born at term. Lastly, we hypothesize that the microbiome in infants born preterm is associated with head circumference growth at term age and improved neurodevelopment at ages 1 to 5.5 years.

## Methods

### Ethical Considerations

The Alberta BLOOM (Begin a Life of Health With Observation and Optimization of the Microbiome) study was approved by the University of Calgary Conjoint Health Research Ethics Board under protocols REB17-1877 (BLOOM–Preterm Neonate Study), REB19-1780 (BLOOM–Long-Term Follow-Up Study), REB20-1442 (BLOOM–Premature Child Study), and REB19-0613 (BLOOM–Brain Magnetic Resonance Imaging substudy). All study procedures were conducted in accordance with the Declaration of Helsinki and applicable institutional and national ethical guidelines.

Written or electronic informed consent was obtained from the parent or legal guardian of each participating child prior to study enrollment. Participants were informed of the study objectives, procedures, potential risks and benefits, the voluntary nature of participation, and their right to withdraw at any time without affecting their medical care. A copy of the signed informed consent form was provided to participating families.

Participant privacy and confidentiality were protected by assigning each participant a unique study identification number used for all study records and biological samples. Personal identifiers were stored separately from research data in secure, access-restricted systems, with access limited to authorized study personnel. Electronic data were stored on secure University of Calgary servers, hard-copy records were maintained in locked facilities, and all biological samples were deidentified prior to analysis and any future sharing for ethically approved research.

Participants received modest compensation to acknowledge their time and participation. Families enrolled shortly after birth received gift cards upon enrollment and completion of the 2-week and 2-month questionnaires, participating children received a small gift at the 3-month corrected age visit, and families attending the 1-year and 3-year follow-up visits received CAD $50 to offset expenses such as parking, transportation, and time. Participants in the neurodevelopment arm also received a CAD $15 gift card following completion of each developmental assessment, CAD $25 gift cards upon enrollment and completion of the 2-week and 2-month questionnaires, participating children received a small gift at the 3-month corrected age visit, and families attending the 1-year and 3-year follow-up visits received CAD $50 to offset expenses such as parking, transportation, and time.

### Cohort Design

#### Overview

**Figure 1.**
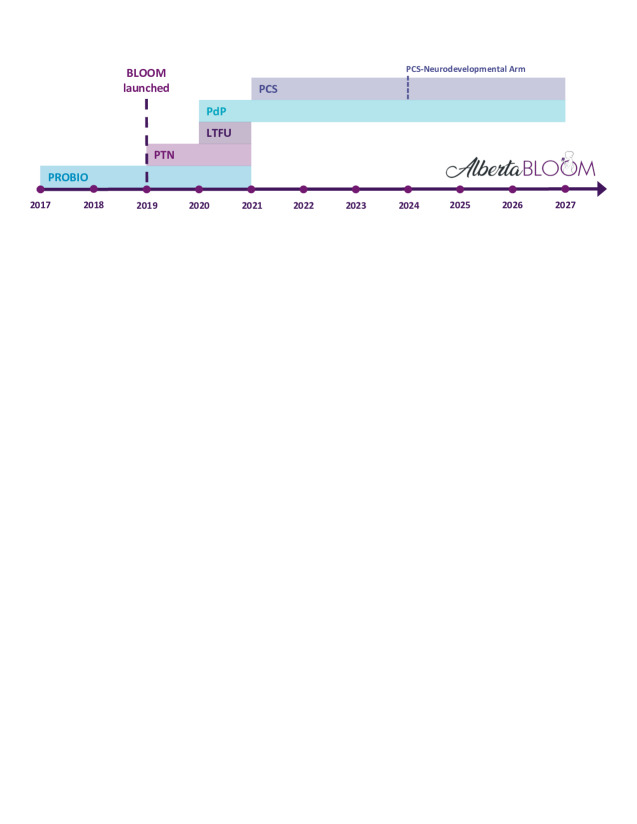
Timeline of BLOOM studies and their associated feeder studies. BLOOM: Begin a Life of Health With Observation and Optimization of the Microbiome; LTFU: Long-Term Follow-Up Study; PCS: Premature Child Study; PdP: Pregnancy During the COVID-19 Pandemic Study; PROBIO: Probiotics and Intestinal Microbiome in Preterm Infants Study; PTN: Preterm Neonate Study.

The Alberta BLOOM research initiative encompasses a suite of individual studies that, together, create a comprehensive cohort of preterm and term infants to map out their microbiome and health trajectories from infancy into early childhood (Figure 1). These BLOOM studies (and associated feeder studies, including PROBIO and PdP; described below) use emerging multiomic technologies to deeply survey the maternal and early childhood microbiome, metabolome (the collection of metabolites in a biological sample), and immune system. The different study arms of BLOOM examine the maternal and infant microbiome at various body sites in association with early postnatal outcomes (eg, sepsis and necrotizing enterocolitis), childhood health outcomes (eg, asthma, allergies, and brain development), and neurodevelopment (eg, cognition, fine and gross motor skills). Additional details of each study are outlined below, and inclusion and exclusion criteria for the different studies are shown in Table 1.

**Table 1.** Inclusion and exclusion criteria of the different BLOOM^a^ studies.

	Inclusion criteria		Exclusion criteria	
	Infants	Caregivers	Infants	Caregivers
PTN^b^	Approached within 72 hours of birth Born ≤31^+6/7^ weeks gestation Expected to survive more than 1 week Admitted to the Foothills Medical Centre level III NICU^c^ No major congenital or chromosomal anomalies	Provide a signed and dated informed consent form Able to speak and understand English 16 years of age or older (if 16‐17 years of age, must be a mature minor and be competent to provide consent) Willing to comply with study procedures and be available for the duration of the study	Congenital gastrointestinal anomalies History of gastrointestinal surgery	Not the legal guardian of the infant In a legal guardianship dispute
LTFU^d^	Born ≤31^+6/7^ weeks gestation Previous participation in the PTN or PROBIO^e^ studies	Consented to contact for future research Provide a signed and dated informed consent form Willing to attend a clinic visit to Alberta Children’s Hospital Able to speak and understand English	Major congenital or chromosomal anomalies	Not applicable
PCS^f^ (arms A/B)	Approached within 14 days of birth or participation in one of the PTN, LTFU, PdP^g^, or PROBIO studies Born ≤36^+6/7^ weeks gestation or previous participation in PdP study (as a term-born reference group) Expected to survive more than 1 week (for preterm infants)	Provide a signed and dated informed consent form Able to speak and understand English 16 years of age or older Willing to comply with study procedures and be available for the duration of the study If recruited as a previous participant of PTN, LTFU, PdP, and/or PROBIO, must be willing to attend a clinic visit(s) at Alberta Children’s Hospital at 1 and 3 years CA^h^ If recruited within 14 days of birth, must reside within the Calgary Metropolitan Region	Major congenital or chromosomal anomalies (including congenital gastrointestinal anomalies) History of gastrointestinal surgery (for infants recruited within 14 days of birth)	Not the legal guardian of the infant In a legal guardianship dispute
PCS (neuro arm)	Born ≤31^+6/7^ weeks gestation Previous or current enrollment in PTN, LTFU, PCS, or PROBIO studies	Provide a signed and dated informed consent form Able to speak and understand English 16 years of age or older Willing to comply with study procedures and be available for the duration of the study	None	Not the legal guardian of the infant In a legal guardianship dispute
MRI^i^	Born ≤31^+6/7^ weeks gestation Be between 2 and 6 weeks CA at the time of the research MRI Previous enrollment in PTN or PCS studies Residing in the Calgary Metropolitan Region	Able to provide consent and understand English	Contraindications to MRI scanning (eg, metal implants, certain dental devices, recent head or chest surgeries) History of intestinal surgery since birth Admitted to hospital at the time of the research MRI Sick (including fever) on the day of the research MRI	Not applicable

a BLOOM: Begin a Life of Health With Observation and Optimization of the Microbiome.

b PTN: Preterm Neonate Study.

c NICU: neonatal intensive care unit.

d LTFU: Long-Term Follow-Up Study.

e PROBIO: Probiotics and Intestinal Microbiome in Preterm Infants Study.

f PCS: Premature Child Study.

g PdP: Pregnancy During the COVID-19 Pandemic Study.

h CA: corrected age.

i MRI: magnetic resonance imaging.

#### BLOOM-PTN and BLOOM-LTFU

BLOOM first began in 2019 with the launch of the BLOOM–Preterm Neonate Study (PTN; ClinicalTrials.gov NCT03840980; registration date February 11, 2019; University of Calgary Conjoint Research Ethics Board CHREB REB17-1877). PTN was designed to study the early-life microbiome of extremely and very preterm infants in relation to their pre- and postnatal microbial exposures. Recruitment of infants born at ≤31^+6/7^ weeks gestation occurred at one tertiary care unit in Calgary, Alberta (Foothills Medical Centre), and continued until 8 weeks postnatally; an additional 3-month CA follow-up time was introduced in 2020. In an effort to follow the longer-term trajectories of these infants, the BLOOM–Long-Term Follow-Up Study (LTFU; ClinicalTrials.gov NCT04641000; registration date November 17, 2020; University of Calgary CHREB REB19-1780) was launched in 2020 to add an additional follow-up time at approximately 1 year CA (between 12 and 24 months CA). BLOOM-LTFU had a particular focus on linking the early-life microbiome to health outcomes, particularly asthma risk (as determined by the Asthma Predictive Index [63]).

#### BLOOM-PCS

The BLOOM–Premature Child Study (PCS; ClinicalTrials.gov NCT05011071; registration date August 11, 2021; University of Calgary REB20-1442) was initiated in 2021 and was designed to integrate the PTN and LTFU studies, while extending the study follow-up until 3 years CA. These studies were developed incrementally to ensure feasibility and optimize study protocols at a smaller scale. Eligibility for BLOOM-PCS was also expanded to include preterm infants born at ≤36^+6/7^ weeks gestation (target: 405 preterm infants total) and those born at term (≥37 weeks’ gestation; target: 130 infants) as a reference group. The study employs ongoing monitoring of recruitment numbers to obtain a balanced distribution of gestation ages across the recruited participants. Together, BLOOM-PTN, BLOOM-LTFU, and BLOOM-PCS constitute the core of the BLOOM studies.

**Figure 2.**
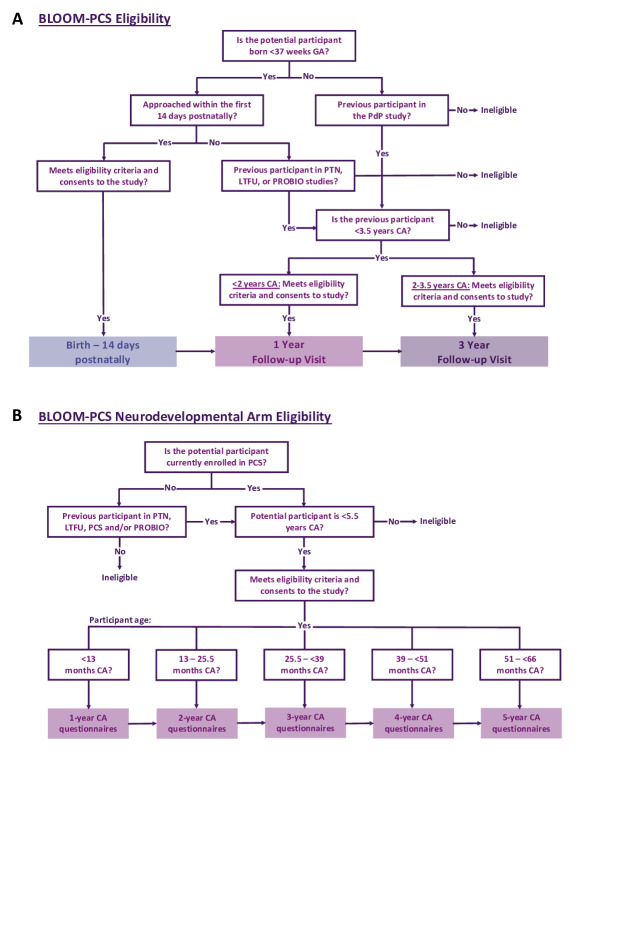
(A) Flow diagrams of eligibility to participate in the BLOOM-PCS study and (B) the BLOOM-PCS Neurodevelopment arm. BLOOM: Begin a Life of Health With Observation and Optimization of the Microbiome; CA: corrected age; GA: gestational age; LTFU: Long-Term Follow-Up Study; PCS: Premature Child Study; PdP: Pregnancy During the COVID-19 Pandemic Study; PROBIO: Probiotics and Intestinal Microbiome in Preterm Infants Study; PTN: Preterm Neonate Study.

BLOOM-PCS includes two recruitment arms depending on the age of the child at enrollment: (1) in-hospital recruitment of preterm infants (arm A) within 14 days of birth and followed until 3.5 years CA and (2) continued follow-up (1‐2 and/or 3‐3.5 years CA; arm B) for infants previously enrolled in BLOOM (PTN and/or LTFU) or from feeder studies (described below), provided they are ≤3.5 years CA (Figure 2A).

#### BLOOM Feeder Studies

Infants could additionally be recruited to arm B (for the later 1- and 3-year follow-up time points) if they previously participated in two studies that collected data and biological samples allowing the study of the microbiome during early life. These are the Probiotics and Intestinal Microbiome in Preterm Infants study from 2017 to 2021 (PROBIO; ClinicalTrials.gov NCT03422562) or the Pregnancy During the COVID-19 Pandemic Study (PdP) launched in 2020 [64,65]. These studies had similar research approaches to BLOOM, allowing for the combination of research efforts and avoiding the collection of duplicate samples and information across studies (Figure 2A). PROBIO was a randomized clinical trial designed to test the effects of a probiotic on the developing gut microbiome of extremely preterm infants (<28 weeks gestation) [64,66]. Infant stool samples in PROBIO were collected in-hospital (first postnatal week, twice during the probiotic intervention [2‐3 weeks and 4-5 weeks postnatally], and after a 2-week washout period) and after the infant was discharged home (6 months CA and 12 months CA). For PdP, recruited infants are born at >35 gestation and followed from birth to 5 years of age [65]. Infants enrolled in PdP had similar samples collected to BLOOM (eg, stool samples); these infants will serve as the term-born reference for BLOOM.

#### BLOOM-PCS Neurodevelopment

An additional third study arm, coined the PCS-Neurodevelopment arm, also recruits infants across the different BLOOM studies (PCS, PTN, LTFU, and/or PROBIO) to monitor their neurodevelopment over the first 5.5 years CA. For the PCS-Neurodevelopment arm, infants are enrolled according to the most age-appropriate questionnaire. For example, parents of infants <11 months CA will be first invited to complete the 12-month questionnaire and will continue in the study until completion at 5 years CA; accordingly, parents of an infant who is 14 months CA will be invited to do the 24-month questionnaire and subsequent time points (Figure 2B).

#### BLOOM-MRI

The Brain MRI in Preterm Infants (BLOOM-MRI) exploratory substudy includes extremely and very preterm infants (born at <32 weeks gestation; enrolled in either PTN or PCS) whose parents consent to them undergoing a brain MRI between 2 and 6 weeks CA. A target enrollment of 20 infants is expected for this pilot study.

### Recruitment

The neonatal intensive care unit (NICU) at Foothills Medical Centre is a Level III NICU, where infants needing the highest level of intensive care are admitted. Pregnant individuals who go into labor at <32 weeks of gestation in the Calgary region are sent to Foothills Medical Centre (if possible) to deliver and infants born <32 weeks gestation are generally admitted to the Foothills Medical Centre NICU immediately following birth. Pregnant individuals who go into preterm labor at >32 weeks gestation may deliver at one of the Calgary hospitals with Level II NICUs, including Peter Lougheed Centre, South Health Campus, or Rockyview General Hospital. For the BLOOM studies, preterm-born participants will therefore be recruited from the NICU at Foothills Medical Centre, in addition to NICUs at the Peter Lougheed Centre (as well as their Pediatrics Unit), South Health Campus, and Rockyview General Hospital.

Study team members will screen admissions to participating hospitals to identify eligible infants born preterm. For all potential study participants, the first approach will be made by a member of the infant’s primary care team with an existing relationship with the child’s caregiver, who will request permission to be approached by a member of the study team. If the caregiver agrees, the research study team member will then approach the family. For BLOOM-LTFU and/or PCS, previous participants of PROBIO and PTN who consented to be contacted for future research will be invited via email and/or telephone. For previous participants of the PROBIO and PdP studies, the first approach will be conducted by a member of the PROBIO or PdP study team, respectively, to ask if they are willing to be contacted by a member of the BLOOM study team to discuss the study.

For the BLOOM-PCS Neurodevelopment arm, a member of the study team will invite previous BLOOM participants (PTN, LTFU, PCS, and/or PROBIO) who provided consent to contact for future research and meet the eligibility criteria. Families will be contacted via email, mail, text, and/or phone for participation invites.

For the BLOOM-MRI substudy, a member of the BLOOM study team will contact the families of potential BLOOM participants (PTN and PCS) between 36 and 40 weeks CA via phone, email, and/or mail to introduce the study and provide an informational pamphlet. Caregivers of potential participants will be subsequently contacted between term CA and up to 6 weeks CA to be invited to join the study and screen for eligibility. Only participants who agreed to be contacted for future studies will be approached.

### Informed Consent

Written or electronic informed consent will be obtained from caregivers to participate in the BLOOM studies. Generally, this consent will be obtained from the birthing parent to access their medical chart and their child’s. The nature of the research study will be explained to caregivers, and a copy of the signed informed consent form and contact information for the investigators will be provided. Caregivers will be advised that their participation is voluntary, and they may withdraw from the study at any time. Once consent is obtained, a study identification number will be assigned, and all study data and subsequent specimen collection will commence. In cases where in-person written consent cannot be obtained at that time (eg, caregiver illness preventing them from visiting the unit, caregiver requires additional time to consider the study and would prefer to be contacted later), a consent to contact form will be completed to allow the research team to contact the participant in-person or via telephone, text, or a secure virtual communication tool, depending on the caregiver’s preference. Informed consent will be obtained thereafter.

### Participant Withdrawal

Participants will be advised that their involvement is voluntary and they may withdraw from the BLOOM studies at any time upon request. Participants who choose to stop participating in this study will have no new data collected or linked to other data that becomes available after their withdrawal date. The study may access, collect, and use data and samples that are available up to the withdrawal date, unless the participating family requests otherwise. Upon withdrawal, participants will be contacted to determine whether they still consent to the completion of all required data extractions from medical databases up to the withdrawal date, and/or permit the study to continue using the data and samples collected during their participation. If participants request that their collected data or samples be removed from the study, any data or samples that have not been processed to remove identifying information will be destroyed. Participants will be made aware that any data or samples that have already been analyzed, processed to remove identifying information, published, or linked with other data cannot be removed.

Investigators may also terminate a study participant’s involvement in the study if a medical condition, event, or situation occurs such that continued participation in the study will not be in the best interest of the participant, or if the participant meets an exclusion criterion (either newly developed or not previously recognized) that precludes further study participation.

### Data Collection

Clinical data are extensively recorded in paper and/or electronic medical records and the Alberta Health Services databases. Data extraction from these electronic resources will be used to ascertain necessary data including, but not limited to, birth characteristics (eg, gestational age at birth, delivery mode, premature rupture of membranes, and small for gestational age at birth), infant acuity scores (eg, Score for Neonatal Acute Physiology-II), infant clinical testing parameters (eg, heart rate, blood pressure, and urine output), maternal history and characteristics (eg, pregnancy complications and socioeconomic status), infant/maternal medications (eg, antibiotics, probiotics, and corticosteroids), infant feeding (eg, daily enteral nutrition, vitamin/mineral supplementation, and breast/chest/bottle feeding), infant diagnoses (eg, necrotizing enterocolitis, sepsis, bronchopulmonary dysplasia, and brain injury), infant procedures (eg, surgery and hospitalizations), child health care visits (eg, vaccinations and weight gain), and laboratory results (eg, bilirubin and complete blood count with differential). A summary of key data collected from BLOOM participants and their families is shown in Table 2.

**Table 2.** Collection of data types across the BLOOM^a^ studies. For the purposes of clarity, medical charts may also include databases through Alberta Health Services.

Data types	Collection timepoint	Data source	Examples
Maternal/birthing parent variables			
Demographics	2 weeks postpartum	Questionnaires	Age, ethnicity, education, sex and gender, socioeconomic status
Clinical factors			
Medical history	Upon enrollment and 2 weeks postpartum	Medical charts, questionnaires	Chronic health conditions, hospitalizations, obstetric history, asthma/allergies
Pregnancy	2 weeks and 2 months postpartum	Medical charts, questionnaires	Infections, medications, vaccinations
Labor	Upon enrollment	Medical charts	Spontaneous labor, delivery mode, rupture of membranes, medications
Nutritional			
Prenatal diet (12 months prior to birth)	2 weeks postpartum	Food frequency questionnaire	Supplements, special diets (eg, vegan), nutritional intakes
Postpartum diet	2 weeks and 2 months postpartum	24-hour dietary recall	Supplements, nutritional intakes
Genetic			
History of preterm birth	2 weeks postpartum	Questionnaires	Maternal, grandmother, siblings
Stress and mental health			
Psychosocial stress	2 weeks postpartum	Questionnaires	The Holmes and Rahe Stress Scale: Stressful Life Events during Pregnancy
Mental health	2 weeks and 2 months postpartum	Questionnaires	History of mental health disorders, Edinburgh Postnatal Depression Scale
Behavioral			
Substance use	2 months postpartum	Questionnaires	Alcohol consumption, tobacco, cannabis
Infant variables			
Clinical factors			
Birth	Upon enrollment	Medical charts	Infant sex, birth anthropometrics, gestational age at birth, APGAR^b^, cord clamping, resuscitation at birth
Medical history	Throughout study	Medical charts, questionnaires	Weekly anthropometrics, laboratory results (eg, complete blood count), morbidities (eg, patent ductus arteriosus, sepsis, retinopathy of prematurity, necrotizing enterocolitis)
Medications	Daily in-hospital and weekly until day 60 if at home; 3 months, 1 year, and 3 years CA^c^	Medical charts, questionnaires	Antibiotics, corticosteroids, caffeine
Interventions	Throughout study	Medical charts	Surgical procedures, respiratory support, hospitalizations
Atopy and wheezing	1 and 3 years CA	Clinic visits, questionnaires	History of wheezing, atopy diagnoses, history of reactions to food or other allergens
Nutritional			
Infant feeding characteristics	Daily in-hospital and weekly until day 60 if at home; 3 months, 1 year, and 3 years CA	Medical charts, questionnaires	Feed type (mother’s milk, pasteurized donor human milk, formula), formula type, parenteral nutrition, fortifiers, breastfeeding, breast pump use, bottle feeding, feeding tubes
Supplements	Daily in-hospital and weekly until day 60 if at home; 3 months, 1 year, and 3 years CA	Medical charts, questionnaires	Probiotics, vitamin D, iron, vitamin A
Childhood diet	1 and 3 years CA	24-hour dietary recall	Supplements, nutritional intakes
Behavioral			
Care and routines	Throughout study	Questionnaires	Kangaroo care, touching, pacifier use, sleeping patterns
Environmental			
Socialization	Throughout study	Questionnaires	Visitors to the home and hospital
Hygiene	3 months, 1 year, and 3 years CA	Questionnaires	Hand washing, home cleaning
Built-in environment	1 and 3 years CA	Questionnaires	Type of dwelling, air cleaner/purifier, carpeting, age of home, pollution nearby
Other environmental factors	Throughout study	Questionnaires	Smoking in the house, pets
Stress-related			
History	Throughout hospitalization	Medical charts	Stressful procedures, needle pokes, invasive ventilation, pain scores
Testing	Throughout study	Clinical assessment, laboratory measures	Brain MRI^d^ (2‐6 weeks CA), hair cortisol (3 months, 1 year, and 3 years CA), urine cortisol (weekly; 1‐60 days)
Immunological			
History	Throughout study	Medical charts, questionnaires	Vaccinations
Testing	1 and 3 years CA	Clinical assessment, laboratory measures	Immune profiling in peripheral blood, skin prick test

a BLOOM: Begin a Life of Health With Observation and Optimization of the Microbiome.

b APGAR: appearance, pulse, grimace, activity, and respiration.

c CA: corrected age.

d MRI: magnetic resonance imaging.

REDCap will also be used to collect and store study data, especially when families have been discharged home and their information is no longer entered into electronic medical charts for data extraction [67,68]. This includes data tracking consent, continued participation in BLOOM, samples collected, study questionnaires, and descriptive data (eg, demographics, medications, and feeding).

All hard copy data will be stored in a locked, secure location with access limited to authorized study personnel. Electronic data are securely stored on university servers with authenticated access for restricted and confidential access. All data (hard copy and electronic) will be retained for a minimum of 15 years after the project is closed and in accordance with the University of Calgary’s Data Retention Policy.

### Data and Sampling Timeline

#### Overview

**Figure 3.**
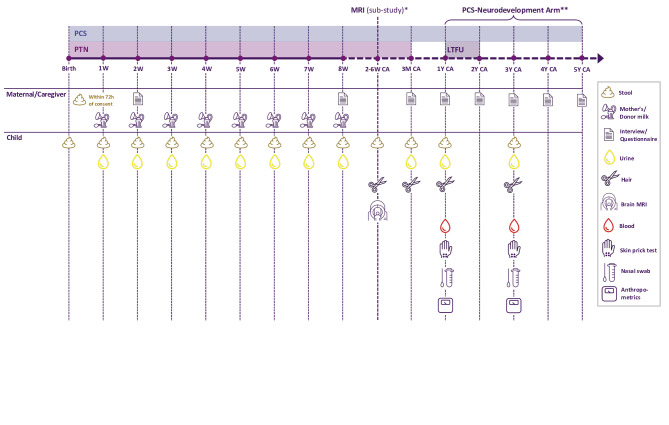
Data and sample collection timeline for the BLOOM studies. The most up-to-date sampling timeline is shown, despite changes in data and sample collection over the course of the BLOOM studies (eg, recent change to collect mothers’ milk weekly in BLOOM-PCS, versus biweekly for PTN and early PCS). *For the 2-6 weeks CA timepoint, samples shown are specific for infants enrolled in the Brain MRI substudy. **The PCS-Neurodevelopmental Arm only includes the neurodevelopmental questionnaires, and not the biological sample collection as part of PCS arms A and/or B. BLOOM: Begin a Life of Health With Observation and Optimization of the Microbiome; CA: corrected age; LTFU: Long-Term Follow-Up Study; MRI: magnetic resonance imaging; PCS: Premature Child Study; PTN: Preterm Neonate Study.

Data and sample collection occurs between birth and 5.5 years CA, depending on the specific BLOOM study that infants and their families are enrolled in. A diagram of the data and sample collection timeline for the BLOOM studies can be found in Figure 3.

#### In-Hospital and Discharge Home (Birth to 60 Days)

##### Data Collection

As described above, information from the infants’ medical charts during their hospital stay will be extracted for those enrolled in BLOOM. Caregivers will also be asked to complete questionnaires at two time points during the first 60 postnatal days: 2 weeks and 2 months. The 2-week study questionnaire (postnatal days 14‐21) includes questions about the parents and family environment (eg, demographics, ethnicity, sexual orientation, gender identity, pregnancy, psychosocial stress, mental and physical health, environment, pets, medical history, and lifestyle factors) and infant care (eg, skin-to-skin). The 2-month study questionnaire (postnatal days 53‐60) expands upon information collected about the parents and family environment (eg, medications, hygiene, home environment, and nutrition) in addition to the infant’s care (eg, feeding, sleep, and health). If an infant is discharged home during the first 60 postnatal days, caregivers will be contacted weekly until 8 postnatal weeks to inquire about the infant’s care, including feeding practices, supplements, medications, and skin-to-skin care.

##### Infant Meconium (First Stool)

The first hours and days following the birth of a preterm infant are often a stressful and challenging time for parents, as their infant is admitted to the NICU and they experience a rapid introduction to the required levels of care. Because of this, and depending upon the severity of the clinical condition during the first days of their child’s life, we believe it would be both unreasonable and impractical to obtain informed consent from parents before collecting the first stool sample (meconium). Therefore, we aim to collect a meconium sample (in a soiled diaper) from all infants in participating NICUs within the first postnatal week who meet the eligibility criteria for BLOOM, allowing families to provide delayed consent without missing the sample. If the meconium sampling occurs prior to consent, the soiled diaper which would otherwise be discarded, will be placed in a sterile study bag by the primary care medical team, labeled with a hospital identification label, and frozen immediately in a freezer (−20 °C) in a secure location within the NICU. If the meconium sample is collected after consent has been obtained, the soiled diaper will be placed in a sealed bag, labeled with a study identification number, and sent to Alberta Precision Laboratories for immediate storage at −20 °C or −80 °C. Samples are collected from Alberta Precision Laboratories by the BLOOM study team within 30 days. If absolutely necessary, collected meconium samples are kept at room temperature for a maximum of 2 hours or in a refrigerator for a maximum of 12 hours prior to freezing. In cases where consent is not obtained, meconium samples will be subsequently discarded by the NICU staff or the BLOOM study team.

##### Maternal Stool

A maternal stool sample will be collected within 72 hours of consent. Participating mothers will be provided with a collection kit and instructions on how to collect and package their stool sample using a toilet hat and study-provided container. Maternal stool samples will then be stored in a refrigerator for up to 12 hours, or frozen in a conventional freezer for a maximum of 4 days, prior to being sent to Alberta Precision Laboratories or the Arrieta Lab for storage at −20 °C or −80 °C, as applicable.

##### Infant Stool (Days 7‐60)

After the meconium sample, infant stools will be collected on a weekly basis starting on postnatal day 7 until 60 days of life, and again at our 3-month, 1-year, and 3-year CA follow-up time points. If consent is obtained after day 7, sample collection will begin from day 14. Diaper changes will be done by the primary care medical team and/or caregiver as per standard of care. As described previously, the soiled diaper will be placed in a sealed bag, labeled with a study identification number, and sent to Alberta Precision Laboratories for immediate storage at −20 °C or −80 °C. Stool samples may be kept at room temperature for a maximum of 2 hours or in a refrigerator for a maximum of 12 hours prior to freezing. If participating infants are discharged to home during the first 60 days postnatally, caregivers will be provided with a child stool collection kit and instructions for home collection. Collected samples will then be couriered from their home to the Arrieta Lab.

##### Infant Urine (Days 7‐60)

Infant urine samples will be collected weekly between postnatal days 7 and 60, followed by single time point collection at 3 months, 1 year, and 3 years CA. Similar to the infant stool samples, urine sampling will begin from day 14 if consent is obtained after postnatal day 7. During a standard of care diaper change, a cotton ball will be added to the new clean diaper. At the next diaper change, the urine-soaked cotton ball will then be placed in a collection container and sent to Alberta Precision Laboratories for immediate storage at −20 °C or −80 °C. Urine samples may be kept at room temperature for a maximum of 1 hour or in a refrigerator for a maximum of 6 hours prior to freezing. If participating children are discharged home during the first 60 days of life, caregivers will be provided with child urine collection kits and instructions for home collection. Collected samples will then be couriered from their home to the Arrieta Lab.

##### Human Milk (Days 7‐60)

Weekly samples of expressed mother’s milk and/or pasteurized donor human milk (total volume 1.5‐10 mL) fed to infants will be collected, as applicable. At the time of preparing feeds, milk that was not needed or used by the infant, and would otherwise be discarded, will be collected for the study. Data regarding the milk expression date, sample collection date, milk type (mother’s milk, pasteurized donor human milk), addition of fortifiers, and donor milk batch numbers will be recorded. Milk samples will be collected in sterile tubes, labeled with a study identification label, and sent to Alberta Precision Laboratories for storage at −20 °C or −80 °C. If participating families are discharged home during the first 60 days of life, caregivers will be provided with breast milk collection kits and instructions for home collection. Collected samples will then be couriered from their home to the Arrieta Lab. Of note, human milk samples for PTN and the early stages of PCS were collected every other week (rather than weekly), starting on postnatal day 14.

### Follow-Up Visit (3 Months CA)

#### Visit Description

For participants enrolled in PCS and in the later stages of PTN, a study visit will be conducted between 2.5 and 3.5 months CA by a trained study team member either by telephone or a secure videoconference call using Doxy.me (as part of the study’s strategy to limit direct contact during the COVID-19 pandemic). During the visit, a study team member will ask interview-style questions about the infant’s general health, nutrition, breastfeeding, pacifier use, and kangaroo care. Questions regarding colds, wheezing, coughs, infections, allergies, hospital and emergency room visits, medications, and vaccinations will also be asked. If necessary, a paper version of the interview-style questions can be provided as well. The following samples will also be collected for the 3-month CA follow-up.

#### Child Stool and Urine

Between 2.5 and 3.5 months CA, caregivers will collect one stool sample (scraped from a soiled diaper into a container) and one urine sample (collected from a cotton ball in a wet diaper) from participating children at home. Samples will then be couriered from the participants’ home to the Arrieta Lab.

#### Child Hair

A child’s hair sample (about 100 strands or a section of approximately 1 cm of hair) will be collected by the caregiver. A hair sample collection kit will be provided, along with a link to an instructional video. Caregivers will be instructed to send the samples upon collection through a courier. Hair strands will then be attached to a piece of clear tape and stored in an envelope at room temperature in a secure location at the Arrieta Lab. Questions about the child’s hair care practices, topical glucocorticoid use, and whether their child was born with hair will also be asked.

### Follow-Up Visits (1 and 3 Years CA)

At 1‐2 years CA and 3‐3.5 years CA, participating children in LTFU and PCS attend follow-up visits at the Alberta Children’s Hospital Pediatric Infectious Diseases Clinic to undergo multiple tests.

#### Stool and Urine Samples

Within ±2 weeks of attending the follow-up clinic visit, caregivers will be asked to collect one stool sample (scraped from a soiled diaper into a container or scooped from a toilet collection hat into a container, depending on toilet training milestones) and one urine sample (collected from a cotton ball in a wet diaper or using a syringe from a toilet collection hat, depending on toilet training milestones) from participating children at home. Samples will then be couriered from the participants’ home to the Arrieta Lab.

#### Nasopharyngeal Swab

During the clinic visit, a study nurse will conduct a nasopharyngeal swab from the participating child’s nose. The swab will be placed into the back of the nostril about 3‐4 cm, depending on the size of the child, and wiped against the inside of the nose.

#### Blood Sample

One blood sample will be collected from participating children. Blood will be drawn with a needle from the arm (about 3‐5 mL or 1 teaspoon) by a trained clinical provider.

#### Child Hair

At the clinic visit, a member of the research team will assist caregivers with collecting a small amount of hair (100 strands or a section of approximately 1 cm of hair) from the nape of the back of the head using pediatric hair safety scissors. If the collection of hair cannot be done during the visit, parents will be provided with instructions on how to collect and courier the sample. Hair strands will be attached to a piece of clear tape and stored in an envelope at room temperature in a secure location at the Arrieta Lab. Caregivers accompanying participating children to the visit will also be asked questions about their child’s hair care practices, topical glucocorticoid use, and whether their child was born with hair.

#### Skin Prick Test

At the study visit, participating children will be tested with the 12 most common allergens for this patient population and observed for signs of allergic sensitization. This will include mold (*Alternaria tenuis*), cat hair, dog epithelium, dust mites (*Dermatophagoides pteronyssinus, Dermatophagoides farinae*), German cockroach, peanut, soybean, egg white, mixed fish, wheat, and cow’s milk. This skin-prick test will include histamine as a positive control and glycerin as a negative control. To conduct the test, a trained care provider will place a drop of solution containing the allergen on the child’s forearm or back. The location of the skin prick test (forearm or back) will be determined based on the most appropriate location for the specific child (eg, size of forearm, skin condition, and parent/guardian preference). Using a small plastic probe or lancet, the care provider will gently prick or scratch the skin to allow a tiny amount of the solution to enter just below the surface. A child will be classified as “atopic” if they develop a wheal ≥2 mm in mean diameter compared to the negative control for any of the allergens tested. If a participant tests positive to glycerin, the wheal size for glycerin will be subtracted from the wheal size of any positive allergen response.

#### Anthropometrics

The child’s weight and length/height will be measured and recorded during the visit. Absolute measurements will also be standardized into *z* scores using the WHO Child Growth Standards [69].

#### Interview

During the visit, caregivers will be asked questions about the child’s general health, medications, atopic dermatitis, hair care, diet, and symptoms/history of wheezing with and without coughing.

#### Questionnaires

We will ask participating caregivers to complete online questionnaires within ±2 weeks of the 1- and 3-year CA follow-up visits. The questionnaires will take about 45‐60 minutes to complete and can be done online over several sessions. Information collected in these questionnaires includes the participating child’s care, nutrition, medical history, health, medication, sleep, feeding environment, hygiene, pets, animal exposure, and caregiver stress.

### Neurodevelopment Arm (1-5.5 Years CA)

This study arm involves the completion of the Ages & Stages Questionnaires, Third Edition (ASQ-3) and the Ages & Stages Questionnaires: Social-Emotional, Second Edition (ASQ:SE-2) by participating parents and their children during the first years of life [70,71]. The ASQ-3 is a developmental screening tool that asks parents a set of questions about their child’s development, including communication, gross motor, fine motor, problem-solving, and personal-social development [70]. The ASQ:SE-2 focuses on screening social and emotional behaviors in young children by asking questions specific to self-regulation, compliance, social-communication, adaptive functioning, autonomy, affect, and interaction with people [71].

Parents will complete the online questionnaires using the study’s secure and validated electronic data capture tool, REDCap. Each questionnaire will take 15‐45 minutes to complete. Accommodations will be made for parents who prefer to complete paper questionnaires. These developmental screening tools are not diagnostic and are intended to answer research questions; however, the results may identify areas that may benefit from a formal assessment by a qualified professional. After the completion of each questionnaire, results will be calculated immediately by REDCap, and caregivers will be presented with the option to view and save their child’s results to learn how they compare to the cutoffs. The number of ASQ-3 and ASQ:SE-2 administered to participants will depend on the child’s CA at enrollment (Figure 2B). The five administration intervals include 1-year (<13 months CA), 2-years (13‐25.5 months CA), 3-years (25.5 to <39 months CA), 4-years (39 to <51 months CA), and 5-years (51 to <66 months CA).

### Brain MRI Substudy (2-6 Weeks CA)

#### Brain MRI

At 2‐6 weeks CA, we will perform brain magnetic resonance imaging (MRI) on a small subset of BLOOM infants (n=20), including anatomical, diffusion-tensor, and resting-state (sleeping) functional MRI. Brain MRI scans will be conducted at Alberta Children’s Hospital, on a research-dedicated 3 Tesla General Electric MR750w MRI scanner. Infants will be scanned during natural sleep using a Med-Vac Immobilizer cushion to reduce the risk of motion. In rare cases, MRI scans of healthy subjects can reveal incidental findings. While the researchers are not trained to identify all abnormalities, and the scans conducted are not the same as clinical MRI scans, if an abnormality is noticed, the scan will be forwarded to a radiologist. The radiologist will examine the scan and determine if follow-up with the participant is necessary.

#### Infant Stool

Caregivers will be asked to collect a stool sample from their infant within 1 week of the MRI. If possible, these samples will be collected during the MRI visit. Otherwise, a home collection kit and collection instructions will be provided during the visit. Collection will involve scraping a small amount of stool from a soiled diaper into a sterile collection container and labeled with a study identification number. Samples will be couriered to the Arrieta Lab for immediate storage at −80 °C. If necessary, stool samples may be kept at room temperature for a maximum of 1 hour, in a refrigerator for up to 12 hours, or in a −20 °C freezer for up to 4 days prior to freezing at −80 °C.

#### Infant Hair

At the MRI visit, a member of the research team will assist parents with collecting a small amount of hair (100 strands or approximately 1 cm) from the nape of the back of the head using pediatric hair safety scissors. Hair strands will be attached to a piece of clear tape and stored in an envelope at room temperature, in a secure location in the Arrieta Lab. Caregivers accompanying participating infants will also be asked questions about infant hair care practices, topical glucocorticoid use, and if the baby was born with hair. If collected at home, a home collection kit and instructions will be provided.

### Participant Retention

To support participant engagement and retention, participating families will receive study reminders about upcoming clinic visits, and required sample collection and questionnaire completion, when applicable. Reminders will be sent via telephone, text, email, and/or the secure REDCap database. A description about the studies is available on the ClinicalTrials.gov and AlbertaBLOOM.ca websites, which participants will be able to access at any time. These websites do not include information that can identify participants; instead, they include resources such as a study overview and sample collection videos, in addition to links to published results.

Participating children (from PTN and PCS-Arm A) will remain in the study when discharged from the hospital, and samples will continue to be collected at home per the study schedule (Figure 3). A courier will transport home-collected samples from the participant’s home to the Arrieta Lab. Weekly phone calls (or via paper or electronic formats, if preferred) will also be made to the participants by the research team to ask about infants’ anthropometrics, feeding, supplements, and medications up to 60 days after birth.

Caregivers will be contacted by the research team to schedule 3-month (PTN/PCS), 1-year (LTFU/PCS), and 3-year (PCS) CA clinic visits, when applicable, as well as complete the ASQ-3 and ASQ:SE-2 (PCS-neurodevelopment arm) at each 1-year timepoint until 5.5 years CA.

### Laboratory Analyses

#### Microbiome and Mycobiome Determination

The microbial profiles of stool and nasopharyngeal swab samples will be determined. For bacteria, stool samples (infant and maternal) will be sequenced using shallow shotgun metagenomics to identify microbial population structures, genes, and metabolic pathways; nasopharyngeal swabs will undergo 16S rRNA gene sequencing using the V4 hypervariable region or shotgun metagenomic sequencing to identify the microbiota present. Fungi will be identified from samples using ITS2 gene sequencing (Illumina MiSeq).

#### Urinary Metabolome

Urine samples will be analyzed for urinary metabolites using enzyme-linked immunosorbent assays (ELISA) to obtain measurements of cortisol, creatinine, and other urinary metabolites at the Drug Safety Lab located at the University of Western Ontario, London, Ontario, Canada. Samples will also undergo untargeted metabolomics to identify host and microbial metabolites at The Metabolomics Innovation Centre in Edmonton, Alberta.

#### Hair Cortisol

Cortisol levels will be measured from hair samples collected at 3 months CA to reflect chronic accumulation of cortisol. Samples will be analyzed at the Drug Safety Laboratory at the University of Western Ontario.

#### Blood Sample Processing

Blood samples collected at the 1- and 3-year follow-up visits will be processed immediately after collection to isolate peripheral blood mononuclear cells (PBMCs) and plasma. About 3-5 mL of whole blood will be collected in an EDTA tube. After centrifugation, the plasma will be transferred to a polypropylene tube and the sediment is used for PBMCs gradient separation. For the plasma, platelets will be removed by centrifugation and the supernatant (plasma) flash frozen. The PBMCs will be isolated using a density gradient medium (Lymphoprep), and washed with 2 mL of phosphate-buffered saline (PBS) and fetal bovine serum (2%). After an additional centrifugation step, the supernatant will be removed and the resulting sediment (PBMCs) mixed with 4 mL of cryoprotective solution and frozen at −80 °C.

#### Immune Analysis of Plasma

Quantitative analysis of cytokines and chemokines will be carried out using the Mesoscale system (MesoScale Discovery). Mesoscale relies on electrochemiluminescence detection of analytes and is considerably more sensitive than ELISA or Luminex multiarray systems. Kits that target a range of pro- and anti-inflammatory cytokines and chemokines will be selected.

#### Immune Cell Analysis From Whole Blood

Approximately 2×10^6^ white blood cells will be stained with purified monoclonal antibodies to determine types and subtypes of immune cells. Samples will then be analyzed using mass cytometry and spectral flow cytometry (CyTOF).

### Long-Term Health Outcomes

#### Asthma and Allergy Risk

Atopy, an important group of immune mechanisms relevant in asthma and other allergic diseases, will be determined by a positive skin prick test to any of the allergens tested. Wheezing history will be captured via questionnaires administered at 1 and 3 years CA based on validated clinical methodology (Asthma Predictive Index), which links assessments at these time points to asthma diagnoses at school age [43,63]. We will consider the presence of atopy and at least one episode of wheezing in the previous year as an atopic-wheeze phenotype, which will be referred to as “asthma risk.”

#### Head Circumference Growth

Head circumference measurements are recorded at birth and throughout the NICU stay, which will be collected from the infant’s medical charts. Absolute measurements will also be standardized into *z* scores using the Fenton Preterm Growth Charts and WHO Growth Standards, as appropriate [69,72].

#### Neurodevelopmental Assessments

Neurodevelopmental outcomes will be measured by using the ASQ-3 and ASQ:SE-2 questionnaires specifically designed to screen for developmental and social-emotional outcomes and risk for delays in young children [70,71].

### Statistical and Data Analyses

#### Sample Size Determination

PTN was initially designed to begin surveying the microbial assembly of preterm infants over time. Most microbiome-based studies of preterm infants at that time included sample sizes between 5 and 50 infants; as such, a target enrollment of 100 infants was chosen. For LTFU, the goal was to determine microbial signatures that distinguished infants with and without atopic wheezing. At the time, the sample size calculation was based on a recently published infant cohort microbiome study [43], which assumed that at least 100 microbial features (operational taxonomic units) would be measured and 10% of them would differ according to atopic wheezing or not; therefore, a total sample size of 84 infants was determined to maintain a false discovery rate below 0.05. For the BLOOM-MRI substudy, this was designed as a pilot study and therefore a convenience sampling method was applied (a total of 20 infants).

For PCS, the study deliverables are focused on the first 3 years of life, based on validated clinical methodology (Asthma Predictive Index), which links assessments at 1 and 3 years with asthma diagnosis at school age [43,63]. Using MetSizeR, assuming at least 200 microbial features will be measured and 10% will differ significantly and median incidence of reported asthma across the gradient of gestational ages studied of 34%, a sample size of 192‐301 is required to maintain a false discovery rate below 5% with 80% power [73]. Assuming 10% dropout, 3.5% deaths, and 12% missed samples, we calculated a final sample size of 405 infants. Using the same assumptions and based on previously generated data from term infants, a sample size of 130 is sufficient to compare microbiome characteristics between term and preterm infants.

For the neurodevelopmental arm of PCS, using microbiome features during the first 3 months of life as the “exposure” and assuming α=.05, we require 390 children to detect a 0.5 standard deviation effect size on the ASQ-3. After accounting for α=.05, and 8% attrition (based on the participants enrolled in the BLOOM-PCS study who have been withdrawn from the study), we will retain 80% power.

#### Microbiome Data Analyses

Sequences will be preprocessed, denoised, and quality filtered prior to downstream analyses. For amplicon sequencing (16S rRNA and ITS2 genes), sequences will be clustered into amplicon sequence variants. For shotgun metagenomics, paired reads will be quality-filtered, human masked, and deduplicated using the Human Microbiome Project’s standards [74]. Diversity metrics (alpha- and beta-diversity; capturing species diversity and overall microbial community structures, respectively) and the relative abundance of microbes will be explored across all sample types. For shotgun metagenomics, gene abundances and their associated pathways will also be determined, in addition to identifying any viruses and phages present. A series of statistical analyses will be used to model microbial outcomes temporally, including linear mixed-effects models for alpha-diversity metrics, permutational multivariate analysis of variance (PERMANOVA) for beta-diversity measures, and multivariate models (eg, zero-inflated negative binomial models in MaAsLin2 [75]; models will be selected depending on their fit of the data) for relative microbial abundances, functions, and metabolites. Key covariates will be adjusted for as fixed effects, including gestational age at birth and infant sex, given their known relationships with microbiome development. Additionally, postnatal week will be included to allow us to test relationships between pre- and postnatal exposures with an infant’s developing gut microbiome over time. *P* values will be adjusted for using Benjamini-Hochberg false discovery rates to account for multiple comparisons. These microbial outcomes will be compared against the term-born reference group, to provide a comparison of the microbiome development of preterm (across different gestational ages) and term infants. Since we have access to the infants’ medical charts, very little clinical and demographic data are missing from the cohort; therefore, we do not plan to fill in missing data through methods such as multiple imputation.

We will also aim to identify microbial taxa, diversity measures, functions, and/or metabolites that predict later health outcomes, including asthma risk and head circumference. Although many approaches can be taken, we aim to use mediation analyses to establish inferred causation between the gut microbiome and future health and disease. Tests of indirect (mediation) effects will be conducted using latent path analyses and a coefficient of products approach. We will also test and include factors that may act as confounders (eg, antibiotic use) in these analyses, as appropriate.

### Biobanking and Data Repository

This cohort is also focused on setting up a biobank and data repository. Therefore, as described in the informed consent form for the study, data and samples will be stored for at least 15 years. The cohort will facilitate storage and reuse of study data through a secure data repository to facilitate future research, in addition to allowing linkage of study data to other datasets. Personal information (eg, name, address, and telephone number) will be removed prior to sharing with other researchers. Researchers who wish to use study data must (1) have their new study approved by an ethics board and (2) sign an agreement that ensures confidentiality and restricts data use to only the approved study. Study data may then be linked with other cohort data to enable further research. This information will be stored on a secure, isolated server, and use of this information will only link data to other datasets.

All samples and any related documentation in the BLOOM studies will be labeled with a study identification number. All biological samples will be stored in freezers in a secure research facility. The labeling of the samples will be done with a study identification number without identifiers such as name, health care number, or initials. The master list, which matches names to identification numbers, will be kept in a separate and secure location that can be accessed only by authorized study personnel. Only study team members will have access to the personal information collected in this study.

## Results

As previously mentioned, recruitment for BLOOM commenced in 2019 with the launch of the BLOOM-PTN study. This quickly expanded in 2020 with BLOOM-LTFU to follow infants until 24 months CA, and again in 2021 with BLOOM-PCS to integrate the objectives of the PTN and LTFU studies, and extend study follow-up to 3 years CA. As of May 2026, we have recruited 245 participants across the different BLOOM studies, and recruitment is ongoing.

Analyses of the BLOOM objectives are currently underway. To begin addressing our primary and secondary objectives, an initial characterization of early-life bacterial and fungal microbiome profiles for the first 105 infants enrolled in BLOOM was published online in December 2025 [76]. This included assessing different infant, maternal, and early-life factors that shape microbiome development in this cohort, such as stress-inducing medical procedures [76].

## Discussion

The BLOOM cohort will be Canada’s most comprehensive observational, prospective cohort study aimed at measuring the microbiome development of preterm-born children. Given the aberrant early-life microbiome observed among preterm infants, and the importance of the gut microbiome for acute and long-term health, this cohort will address a significant research priority by characterizing the microbial development patterns of preterm infants in the first 3 years and correlating it with their asthma and allergy risk, head growth, neurodevelopment, and overall health. Furthermore, by including the full spectrum of prematurity, in addition to a term-born reference cohort, findings from these studies will be more generalizable to the preterm population.

Results from this cohort hold promise in providing novel insights into how the early-life microbiome develops in preterm-born children and how various pre- and postnatal factors shape these patterns of development. In particular, the integration of microbial data with detailed, longitudinal clinical data and biological measures (eg, metabolic and immune profiles) will provide a nuanced and in-depth assessment of microbial development in preterm infants and factors associated with these dynamics. These findings hold considerable weight in influencing medical practice, parenting choices, consumer product regulation, and policy development. More specifically, this may include purchasing behaviors, decisions about childbirth and delivery, diet, breastfeeding, cleaning products used in homes, owning a family pet, and dealing with stress.

We also anticipate this research will help identify epidemiological risk factors and biomarkers that can provide diagnostic value or predictive information for disease prevention. For instance, our work will be among the first to explore prematurity-driven dysbiosis as a contributing factor to microbiome-related health outcomes such as asthma and allergies. Until now, reduced lung function in this pediatric population is considered an unavoidable result of developmental immaturity and structural lung damage. Studying how the microbiome may play a role in asthma development in preterm infants, as it does in infants born at term, may open the possibility to prevent this disease through microbial therapies or interventions. In addition, our investigation into how the microbiome, and specifically the loss of normal microbiome-derived metabolites, sculpts the long-term immune responses could herald the development of metabolite-based interventions that could ensure a more balanced development of innate and adaptive immune cell subsets. Our research also holds potential to inform the design of microbial therapeutics (eg, preventative next-generation probiotics) for later clinical testing, thereby supporting bench-to-bedside discovery and clinical translation.

Our cohort will provide critical insight into how the gut microbiome may drive neurodevelopment in infants born preterm—a concept that is poorly understood, despite the pressing burden of neurodevelopmental impairments in this population [52-60]. Our work will determine if the well-established link between head circumference growth and neurodevelopmental delay can be explained by microbiome immaturity or other microbiome-related factors in infants born preterm. These findings could advance our understanding of neurodevelopmental delays and provide a basis for improved screening, treatment practices, and microbial therapeutics.

Lastly, a comprehensive biobank of thousands of biological samples and corresponding clinical data will be constructed as part of BLOOM. This tremendous undertaking will enable future collaborators to access samples and data to further our study of early-life microbiomes, preterm infant development, and long-term health outcomes.

In conclusion, this innovative cohort is well-positioned to increase our current knowledge of microbiome development in preterm infants and its role in shaping disease risk and neurodevelopment.

## Supplementary material

10.2196/95658 Peer Review Report 1 Peer Review Report by Research Teams Committee, Canadian Institutes of Health Research (CIHR).

## Abbreviations

ASQ: Ages & Stages Questionnaires
BLOOM: Begin a Life of Health With Observation and Optimization of the Microbiome
C-section: cesarean section
CA: corrected age
CHILD: Canadian Health Infant Longitudinal Development Study
ELISA: enzyme-linked immunosorbent assay
LTFU: Long-Term Follow-Up Study
MRI: magnetic resonance imaging
NICU: neonatal intensive care unit
PBMC: peripheral blood mononuclear cell
PBS: phosphate-buffered saline
PCS: Premature Child Study
PdP: Pregnancy During the COVID-19 Pandemic Study
PERMANOVA: permutational multivariate analysis of variance
PROBIO: Probiotics and Intestinal Microbiome in Preterm Infants Study
PTN: Preterm Neonate Study
