# Supplementary material for: Exploring the Early-Life Microbiomes of Preterm Infants and Their Childhood Health Outcomes (The BLOOM Study): Protocol for a Prospective, Observational Cohort Study
Source: JMIR Res Protoc. 2026 Aug 18;15:e95658. doi: 10.2196/95658 (PMC13484930; doi:10.2196/95658)
Supplement: Supplementary file 1 [file resprot-v15-e95658-s001.pdf]

November/Novembre 28, 2019

DC0190GP

Canadian Institutes of Health Research / Instituts de recherche en santé du Canada

Notice of Decision / Avis de décision

Application Number/Numéro de la demande: 429655

Committee Code/Code du comité: MRT

Applicants/Candidats: Dr. Marie Claire Arrieta Mendez, Dr. Meghan Azad, Dr. Gerald Frank Giesbrecht, Dr. Steven William Kembel, Dr. Anita L Kozyrskyj, Dr. Morgan Gavel Ira Langille, Dr. Kelly Marshall McNagny, Dr. Laura Kuhn Sycuro

With/Avec: Dr. B. Alshaikh, Dr. C. Field, Dr. D. Figeys, Dr. J. Kellner, Dr. C. Lebel, Dr. L. Leijser, Dr. K. McCoy, Dr. T. Tompkins

Institution paid/ Établissement payé: University of Calgary

Title/Titre: Contribution of the microbiome to asthma risk in premature infants

Primary Inst./ Inst. principal: Human Development, Child and Youth Health / Développement et santé des enfants et des adolescents

Other Related Inst./ Autres inst. connexes: Circulatory and Respiratory Health / Santé circulatoire et respiratoire; Infection and Immunity / Maladies infectieuses et immunitaires

Competition Outcome/Résultats du concours: Team Grant: Canadian Microbiome Initiative 2: Research Teams / Subvention d'équipe : Initiative canadienne microbiome 2 : Équipes de recherche

September/Septembre 24, 2019

Number in competition/Nbre de demandes dans le concours: 19 (includes 1 application in the IHDCYH pool and 1 application in the JDRF pool)

Number approved/Nbre de demandes approuvées: 7 (includes 1 application in the IHDCYH pool and 1 application in the JDRF pool)

Decision on your application/ Décision sur votre demande: Approved / Approuvée

Average annual amount/ Montant annuel moyen: $399,988

Term/Durée: 5 **yrs/ans** 0 **months/mois**

Peer Review Committee Recommendation, for your information and use/ Recommandation du comité d'examen par les pairs, pour fins d'information et d'utilisation:

Committee/Comité: Team Grant : Canadian Microbiome Initiative 2: Research Teams / Subvention d'équipe : Initiative canadienne microbiome 2 : Équipes de recherche

Application rank within the competition/ Rang de la demande dans ce concours: 3

Percent Rank Within the Competition/ Rang en pourcentage au sein du concours: 21.05%

Rating/ Cote: 4.31

Recommended average annual amount/ Montant annuel moyen recommandé: $355,225

*** Applications receiving a score of less than 3.5 on any evaluation criteria will not be considered for Funding. / Les demandes qui ont reçu une note inférieure à 3.5 pour n'importe quel des critères d'évaluation ne sont pas admissibles.

Additional Funding Opportunities/ Opportunités de financement additionnelles	Decision/ Décision	Rank/ Rang	Competition Code/Cote de concours
Team Grant: Canadian Microbiome Initiative 2: Research Teams - IHDCYH Pool/Sub d'équipe: Initiative can. microbiome 2 : Équipes de recherche - Classe IDSEA	Not Approved/ Non Approuvée	3/10	201909MT2

This document is for information only. Official payment is stated on the CIHR Authorization for Funding.
Document à titre d'information seulement. Le paiement officiel est indiqué sur l'autorisation de financement des IRSC.

Canadian Institutes of Health Research
160 Elgin Street, 9th Floor
Address Locator 4809A
Ottawa, Ontario K1A 0W9

Instituts de recherche en santé du Canada
160, rue Elgin, 9e étage
Indice de l'adresse 4809A
Ottawa (Ontario) K1A 0W9

Institute of Aging
Institute of Cancer Research
Institute of Circulatory and Respiratory Health
Institute of Gender and Health
Institute of Genetics
Institute of Health Services and Policy Research
Institute of Human Development and Child and Youth Health
Institute of Indigenous Peoples' Health
Institute of Infection and Immunity
Institute of Musculoskeletal Health and Arthritis
Institute of Neurosciences, Mental Health and Addiction
Institute of Nutrition, Metabolism and Diabetes
Institute of Population and Public Health

Institut du vieillissement
Institut du cancer
Institut de la santé circulatoire et respiratoire
Institut de la santé des femmes et des hommes
Institut de génétique
Institut des services et des politiques de la santé
Institut du développement et de la santé des enfants et des adolescents
Institut de la santé des Autochtones
Institut des maladies infectieuses et immunitaires
Institut de l'appareil locomoteur et de l'arthrite
Institut des neurosciences, de la santé mentale et des toxicomanies
Institut de la nutrition, du métabolisme et du diabète
Institut de la santé publique et des populations

November 28, 2019

Dr. Marie Claire Arrieta Mendez
HRIC 4AC66
3330 Hospital Drive NW
University of Calgary
Calgary, Alberta T2N 4N1

Dear Dr. Arrieta Mendez:

On behalf of the Canadian Institutes of Health Research (CIHR) we are pleased to inform you that your recent application submitted to the Team Grant: Canadian Microbiome Initiative 2: Research Teams competition entitled, "Contribution of the microbiome to asthma risk in premature infants" has been approved for funding. Documentation pertaining to the review of your application can be found through ResearchNet. Please note that your Authorization for Funding will follow in the mail.

As CIHR does not notify co-applicants of the decision, we ask that you inform those individuals involved, along with their research institutions (if different from your own), of the outcome of this application.

Should you require additional information, please contact the CIHR Contact Centre at support-soutien@cihr-irsc.gc.ca. Please do not contact the officers or members of the peer review committee.

Congratulations on your success in this competition.

Sincerely,

C. Leneï

Chaidwick Leneïs
Manager, Program Design and Delivery
Research Programs Portfolio

489907-201909MRT-MRT-429655-I012-168024-351A

Canada

CIHR IRSC
Discoveries for life / Découvertes pour la vie

Canadian Institutes of Health Research
160 Elgin Street, 9th Floor
Address Locator 4809A
Ottawa, Ontario K1A 0W9

Instituts de recherche en santé du Canada
160, rue Elgin, 9e étage
Indice de l'adresse 4809A
Ottawa (Ontario) K1A 0W9

Institute of Aging
Institute of Cancer Research
Institute of Circulatory and Respiratory Health
Institute of Gender and Health
Institute of Genetics
Institute of Health Services and Policy Research
Institute of Human Development and Child and Youth Health
Institute of Indigenous Peoples' Health
Institute of Infection and Immunity
Institute of Musculoskeletal Health and Arthritis
Institute of Neurosciences, Mental Health and Addiction
Institute of Nutrition, Metabolism and Diabetes
Institute of Population and Public Health

Institut du vieillissement
Institut du cancer
Institut de la santé circulatoire et respiratoire
Institut de la santé des femmes et des hommes
Institut de génétique
Institut des services et des politiques de la santé
Institut du développement et de la santé des enfants et des adolescents
Institut de la santé des Autochtones
Institut des maladies infectieuses et immunitaires
Institut de l'appareil locomoteur et de l'arthrite
Institut des neurosciences, de la santé mentale et des toxicomanies
Institut de la nutrition, du métabolisme et du diabète
Institut de la santé publique et des populations

November 28, 2019

Dr. Marie Claire Arrieta Mendez
HRIC 4AC66
3330 Hospital Drive NW
University of Calgary
Calgary, Alberta T2N 4N1

Dear Dr. Arrieta Mendez:

On behalf of the Canadian Institutes of Health Research (CIHR), I wish to congratulate you on your success in the recent CIHR funding competition!

As health researchers, we are united by a common goal: to improve the health and well-being of Canadians and people throughout the world. Through CIHR, the Government of Canada provides vital support to Canadian researchers, spanning the tightly linked pillars of health research, with the ultimate goal of improving health for all Canadians. As a recipient of this funding, you are now part of this endeavour.

As you are aware, the evaluation of your grant application was made possible thanks to peer reviewers who generously volunteer their time to support the Canadian health research enterprise. As a CIHR-funded researcher, your knowledge and expertise are invaluable to this process. If you are not already a peer reviewer, I would like to take this opportunity to invite you to consider becoming a member of the College of Reviewers. Please visit www.cihr-irsc.gc.ca/e/49923.html to learn about the eligibility criteria and how to apply for membership.

As health researchers, we are living at a time when science is under careful scrutiny, and it has become more important than ever to ensure that our work is clearly understood. As such, I would ask that you recognize your CIHR funding when delivering presentations and communicating about your research, and that you continue to advocate for the critical importance of research in advancing the health of Canadians.

Once again, please accept my congratulations on this impressive achievement and my best wishes for success in all your endeavours. I look forward to following the progress of your research.

Sincerely,

Michael J. Strong, MD, FRCPC, FAAN, FCAHS
President

489908-201909MRT-MRT-429655-I012-168024-CONGR

Review Type/Type d'évaluation: Committee Member 1/Membre de comité 1
Name of Applicant/Nom du chercheur: Arrieta Mendez, Marie Claire
Application No./Numéro de demande: 429655
Agency/Agence: CIHR/IRSC
Competition/Concours: 2019-09-24 Team Grant: Canadian Microbiome Initiative 2: A Focus on Function and Translation Research Teams/Subvention d'équipe : Équipes de recherche de l'Initiative canadienne du microbiome 2 : fonction et application des connaissances
Committee/Comité: Team Grant : Canadian Microbiome Initiative 2: Research Teams/Subvention d'équipe : Initiative canadienne microbiome 2 : Équipes de recherche
Title/Titre: Contribution of the microbiome to asthma risk in premature infants

---

Assessment/Évaluation:

Evaluation Criteria

The hypothesis of this proposal is that the microbiome of preterm infants differs from that of full term infants and increases risk of atopy and asthma through impact on immune and neuroimmune mechanisms. The objectives of the proposal are to: 1) define the microbiome, familial, stress and immune factors associated with atopic wheeze in 405 children from the BLOOM cohort study at 1 and 3 years of age; 2) determine microbiome features distinguishing between preterm and full term children at risk of asthma; and 3) test the causality and immune/neuroimmune mechanisms in a mouse asthma model.

1. Approach

Strengths: The availability of the BLOOM cohort for study of preterm infants is a major strength and preliminary data indicates that microbiome in late preterm (35-37 week) infants is altered from full term infants. However, no information is provided on the atopic or asthma relative risk between late preterm and full term (>37 week) infants. For the altered microbiome to have relevance. Data is provided on extremely preterm (<29 week) infant microbiome giving two major bacterial clusters and one fungal cluster which apparently corresponds to known results in these infants. The study will obtain a significant amount of information including health history, behavioural factors including some stress analyse**s and immune profiling. Modelling should allow investigators to define risk factors among bacteria taxa, diversity indices, metabolites, mode of birth, GA, antibiotic use, and immune phenotypes. The major advantage would defining how microbiomes alter over time. Aim 3 will inoculate germ-free neonates with FMT from extreme, late preterm and full term infants then treated with antibiotic for 7 days and some treated with maternal separation for stress induction. Gut immune profiling, asthma induction and microglial profiling will be performed. Although not stated, one assumes that gut microbiome will be evaluated to confirm residual colonization resembles human infants and that stress indicators will be measured.**

Weaknesses: Exposure to foreign antigens in extreme preterm infants ought to result in central tolerance and major disruption of T cell repertoire having a major impact on life-long microbiome composition. While the impact would be well-recognized for the adaptive immune response, even the innate immune response (gamma-delta T cells, NKT cells, possibly NK cells, ILC) can be altered/tolerized at these early time points impacting gut-associated immunity. Even if specific bacterial taxa are identified in preterm infants, the major question is whether it is the bacteria or the state of the immune system causing increased atopic risk. CyTOF is performed for immune profiling but it is unclear whether this will distinguish the potential immune dysfunctions in extreme preterm infants. Aim 3B is the weakest of the Aims. The studies will use the Oligo-MM mice containing stable low diversity mouse gut bacterial colonies which allow colonization by other bacteria. These mice will be colonized with select bacterial taxa implicated as relevant. It is not clear how many different bacteria may need to be tested, whether single bacteria taxa independently promote risk or

**Review Type/Type d'évaluation:**	Committee Member 1/Membre de comité 1
**Name of Applicant/Nom du chercheur:**	Arrieta Mendez, Marie Claire
**Application No./Numéro de demande:**	429655
**Agency/Agence:**	CIHR/IRSC
**Competition/Concours:**	2019-09-24 Team Grant: Canadian Microbiome Initiative 2: A Focus on Function and Translation Research Teams/Subvention d'équipe : Équipes de recherche de l'Initiative canadienne du microbiome 2 : fonction et application des connaissances
**Committee/Comité:**	Team Grant : Canadian Microbiome Initiative 2: Research Teams/Subvention d'équipe : Initiative canadienne microbiome 2 : Équipes de recherche
**Title/Titre:**	Contribution of the microbiome to asthma risk in premature infants

Assessment/Évaluation:

protection, or whether synergy between bacteria taxa might be relevant. Combinations would be used if relevant but how these might be determined is not stated. The induction of mucosal tolerance presumably looks for taxa promoting protection or reduced asthma but whether this is really tolerance is not studied. As for LPS tolerance, most of the cited publications induced high dose E. coli LPS tolerance directly in mouse autoimmunity models which is different from assuming that microbiota would naturally produce a tolerogenic state. The closest reference to supporting the investigator's hypothesis is reference #158 but this basically compared two human populations differing in autoimmunity susceptibility (Finland and Russia) and having predominantly distinct bacteria tax as LPS sources (E. coli, B. dorei LPS) and in which E. coli LPS correlates with autoimmunity not protection. There might be other characteristics of the B. dorei LPS leading to protection.

2. Team

Strengths: Dr. Arrieta Mendez is an Assistant Professor in Physiology-Pharmacology and Pediatrics, publishes on experimental probiotics and has excellent grant funding. She established the BLOOM cohort. Drs. Kozyrskyj, Azad, and McNagny have expertise in both microbiome and asthma research using animal models and in humans. Drs. Kembel, Sycuro, Langille, and Figeys provide expertise in statistical and computational analysis. The team involves 8 institutions from across Canada showing broad geographical distribution.

The international advisory board is outstanding in their expertise and access to further cohorts and research material if needed.

Weaknesses: none

3. Envirionment

Strengths: The facilities are excellent and sufficient to the proposed work.

Weaknesses: none

4. Potential Impact

Strengths: Asthma is a significant health risk both in Canada and globally. Preterm infants have a significantly increased risk .The reasons are not absolutely clear but there may be multiple factors including life-long alterations in their microbiome which contribute to the risk. The studies in Aim 1 should define many of the lifestyle, clinical and behavioural factors impacting increased risk possibly associating this with microbiota and the HPA.

Weaknesses: There are likely to be other considerations not addressed in the proposal. The most obvious of these is that exposure of extreme preterm infants to microbes should perturb immune development such as tolerance induction either of adaptive immunity alone or of both adaptive and innate effectors. There is also the question whether exposure to normal levels of microbiota would actually induce LPS tolerance. The tolerance cited by the PI, with one exception, reflects the

**Review Type/Type d'évaluation:**	Committee Member 1/Membre de comité 1
**Name of Applicant/Nom du chercheur:**	Arrieta Mendez, Marie Claire
**Application No./Numéro de demande:**	429655
**Agency/Agence:**	CIHR/IRSC
**Competition/Concours:**	2019-09-24 Team Grant: Canadian Microbiome Initiative 2: A Focus on Function and Translation Research Teams/Subvention d'équipe : Équipes de recherche de l'Initiative canadienne du microbiome 2 : fonction et application des connaissances
**Committee/Comité:**	Team Grant : Canadian Microbiome Initiative 2: Research Teams/Subvention d'équipe : Initiative canadienne microbiome 2 : Équipes de recherche
**Title/Titre:**	Contribution of the microbiome to asthma risk in premature infants

Assessment/Évaluation:

difference between high dose LPS exposure on farms versus in urban areas, not what may be more subtle differences in microbiome compositions in infants.

**Review Type/Type d'évaluation:**	Committee Member 2/Membre de comité 2
**Name of Applicant/Nom du chercheur:**	Arrieta Mendez, Marie Claire
**Application No./Numéro de demande:**	429655
**Agency/Agence:**	CIHR/IRSC
**Competition/Concours:**	2019-09-24 Team Grant: Canadian Microbiome Initiative 2: A Focus on Function and Translation Research Teams/Subvention d'équipe : Équipes de recherche de l'Initiative canadienne du microbiome 2 : fonction et application des connaissances
**Committee/Comité:**	Team Grant : Canadian Microbiome Initiative 2: Research Teams/Subvention d'équipe : Initiative canadienne microbiome 2 : Équipes de recherche
**Title/Titre:**	Contribution of the microbiome to asthma risk in premature infants

Assessment/Évaluation:

Intro

This proposal aims to address the role of the microbiome in asthma in preterm infants. Asthma is a huge issue in Canada and beyond. A causal link between the microbiome, dysbiosis and asthma has been established, including a recent publication by the applicants, who are experts in this field. Premature infants show a significant amount of microbiome dysbiosis for various reasons yet asthma has never been studied in premature infants, who are at highest risk of developing asthma. This study aims to identify how prematurity-driven dysbiosis impacts asthma risk via immune and neuroimmune mechanisms. This study will leverage the BLOOM study (longitudinal study of premature infants and mothers). Objectives are to define the microbiome of pre-term infants and immune factors of these infants using standard microbiome analysis techniques, along with functional microbiome analysis of some samples (metagenomics and metaproteomics) and monitoring of immune function (antibody levels, cytokine production, cell analysis) and neuroimmune function (measuring cortisol, MRI follow up in some cases); determine which microbiome features mediate asthma risk (and how this is different from full-term babies); and test causality of mechanisms using GF mice

Impact of Research

This research will develop new knowledge on the microbial makeup of preterm infants, and correlations between these taxa and the risk of asthma. While new preventative and therapeutic interventions will not be a direct focus of this work, it may lend new insight to contribute to future interventions for microbiome alterations in preterm babies.

The team will work with industry including probiotic developers who may ultimately translate the data from this study into preventative probiotics for asthma prevention. Team has an established partnership with Lallemand which is a strength.

Originality of Research proposal

The team is exploiting novel methods including a focus not only on the microbiome, but on other factors including the patient immune and neuroimmune response, and functional microbiome analysis using metaproteomics. In each case, relevant experts in each of these approaches is well integrated as part of the team. A strength of this proposal is integrating microbiome analysis with immune and neurological impacts, and relevant experts in these fields.

Additionally, a strength is the inclusion of computational biology and bioinformatic analysis and the use of different modeling techniques to identify microbial species that best predict asthma outcomes, and models to determine the association between variables (gestational age etc) and asthma risk or immune phenotypes.

**Review Type/Type d'évaluation:**	Committee Member 2/Membre de comité 2
**Name of Applicant/Nom du chercheur:**	Arrieta Mendez, Marie Claire
**Application No./Numéro de demande:**	429655
**Agency/Agence:**	CIHR/IRSC
**Competition/Concours:**	2019-09-24 Team Grant: Canadian Microbiome Initiative 2: A Focus on Function and Translation Research Teams/Subvention d'équipe : Équipes de recherche de l'Initiative canadienne du microbiome 2 : fonction et application des connaissances
**Committee/Comité:**	Team Grant : Canadian Microbiome Initiative 2: Research Teams/Subvention d'équipe : Initiative canadienne microbiome 2 : Équipes de recherche
**Title/Titre:**	Contribution of the microbiome to asthma risk in premature infants

Assessment/Évaluation:

A causal link with microbiome will be assessed with gnotobiotic mouse studies using pooled stool from different premature infant groups, as well as using targeted bacterial/fungal strains identified as having a potentially causal link. It is not immediately clear how many different taxa of organisms will be tested, and /or if they will be tested together as a community or as independent strains of interest.

Research approach

Hypothesis and objectives are extremely clear and well defined. Objectives are the define the microbiome of pre-term infants and immune factors of these infants, determine which microbiome features mediate asthma risk (and how this is different from full-term babies), and test causality of mechanisms using GF mice

Clear hypothesis based on previous and preliminary data. Preliminary data from the applicants shows Streptococcus (not previously associated with asthma) abundance is altered in preterm infants and associated with asthma risk, and indicating they may be able to identify other factors that impact asthma risk.

Involvement with BLOOM study a significant asset, which includes extensive sampling of infant and maternal samples in weeks/months following birth, and will include neuroimmune markers as well (cortisol and MRI scans in some cases). Infants will be followed for 3 years.

Using appropriate modeling techniques to identify bacterial and fungal composition of premature infant gut. Inclusion of prokaryotic and eukaryotic organisms in analysis is a strength. Functional microbiome analysis (metagenomics and metaproteomics) of select samples is also a strength of this proposal.

A strength of this proposal is the excellent integration of relevant modelling and statistical techniques, and demonstration of ability to apply these methods based on previous work by the applicants.

More details on the mouse model applied, and how it represents a model for human asthma would be beneficial, as currently it lacks detail as to how well this particular model can represent premature infant asthma, and how asthma severity will be measured in the mice.

Sex and gender addressed though stratified and regression models. Also collecting data on physical touch between parents and children and the use of male and female gnotobiotic mice.

Team

This proposal has a strong team of researchers from multiple institutions (Calgary, Manitoba, UQAM, Alberta, UBC), and across a wide variety of specialities (microbiology, systems biology, immunology,

**Review Type/Type d'évaluation:**	Committee Member 2/Membre de comité 2
**Name of Applicant/Nom du chercheur:**	Arrieta Mendez, Marie Claire
**Application No./Numéro de demande:**	429655
**Agency/Agence:**	CIHR/IRSC
**Competition/Concours:**	2019-09-24 Team Grant: Canadian Microbiome Initiative 2: A Focus on Function and Translation Research Teams/Subvention d'équipe : Équipes de recherche de l'Initiative canadienne du microbiome 2 : fonction et application des connaissances
**Committee/Comité:**	Team Grant : Canadian Microbiome Initiative 2: Research Teams/Subvention d'équipe : Initiative canadienne microbiome 2 : Équipes de recherche
**Title/Titre:**	Contribution of the microbiome to asthma risk in premature infants

Assessment/Évaluation:

computational biology…), and includes clinicians and epidemiologists.
The team has a strong track record in microbiome research and a very strong publication record and funding, and are well equipped to perform this study
Inclusion of ECIs alongside mid/senior career researchers in leadership roles is a strength - this proposal is led by an ECI with a strong track record for publications and grant funding.
There is very clear and strong expertise in all of needed areas, from establishing clinical cohorts and conducting clinical studies (and the groups BLOOM study), strong expertise in microbiome research and relevant bioinformatic analysis, gnotobiotic mouse models, statistics etc.
A plan is in place to ensure communication within the group and a mentorship plan based on embedding ECIs with senior researchers within research sub groups.
Team includes an international advisory committee of prominent microbiome researchers and a sex and gender champion.

Review Type/Type d'évaluation: Committee Member 3/Membre de comité 3
Name of Applicant/Nom du chercheur: Arrieta Mendez, Marie Claire
Application No./Numéro de demande: 429655
Agency/Agence: CIHR/IRSC
Competition/Concours: 2019-09-24 Team Grant: Canadian Microbiome Initiative 2: A Focus on Function and Translation Research Teams/Subvention d'équipe : Équipes de recherche de l'Initiative canadienne du microbiome 2 : fonction et application des connaissances
Committee/Comité: Team Grant : Canadian Microbiome Initiative 2: Research Teams/Subvention d'équipe : Initiative canadienne microbiome 2 : Équipes de recherche
Title/Titre: Contribution of the microbiome to asthma risk in premature infants

---

Assessment/Évaluation:

Impact of the Research

Common disease (1 in 8 children)

Cohort design following until age 3

Pilot data

Atopy is indirect correlate of asthma

405 infants

Metagenomic and metaproteomic assessment

maternal and fetal,

Microbiome features which mediation asthma risk, not only cause asthma

Fecal transplants in mice

Builds on ongoing cohort study

2. Originality of the Proposal

First study in premature infants

Biweekly sampling

Cortisol and MRI sampling

Good pilot data

Shallow shotgun and ITS2 sequencing (strain level)

**Review Type/Type d'évaluation:**	Committee Member 3/Membre de comité 3
**Name of Applicant/Nom du chercheur:**	Arrieta Mendez, Marie Claire
**Application No./Numéro de demande:**	429655
**Agency/Agence:**	CIHR/IRSC
**Competition/Concours:**	2019-09-24 Team Grant: Canadian Microbiome Initiative 2: A Focus on Function and Translation Research Teams/Subvention d'équipe : Équipes de recherche de l'Initiative canadienne du microbiome 2 : fonction et application des connaissances
**Committee/Comité:**	Team Grant : Canadian Microbiome Initiative 2: Research Teams/Subvention d'équipe : Initiative canadienne microbiome 2 : Équipes de recherche
**Title/Titre:**	Contribution of the microbiome to asthma risk in premature infants

Assessment/Évaluation:

Excellent exploration of co-variables and stress physiology

3. Research Approach

Good sample size calculation

Includes anticipated challenges and mitigation strategies

Unique analysis methods

Strong international partnership and financial contributions

4. Team

7 PI’s from 5 universities, 7 advisors and a patient

Review Type/Type d'évaluation: SO Notes /Notes de l'agent scientifique
Name of Applicant/Nom du chercheur: Arrieta Mendez, Marie Claire
Application No./Numéro de demande: 429655
Agency/Agence: CIHR/IRSC
Competition/Concours: 2019-09-24 Team Grant: Canadian Microbiome Initiative 2: A Focus on Function and Translation Research Teams/Subvention d'équipe : Équipes de recherche de l'Initiative canadienne du microbiome 2 : fonction et application des connaissances
Committee/Comité: Team Grant : Canadian Microbiome Initiative 2: Research Teams/Subvention d'équipe : Initiative canadienne microbiome 2 : Équipes de recherche
Title/Titre: Contribution of the microbiome to asthma risk in premature infants

---

Assessment/Évaluation:

Strengths:
Asthma represents a significant health risk and burden on society, and a causal link between microbiome, dysbiosis and asthma has been established.

The investigators are experts and leaders in this field. Access to the BLOOM cohort, established by the nominated PI (who is also an ECI), was viewed as an obvious strength.

Aim1 in particular was viewed as a clear strength of the proposal

The team is geographically diverse. Co-PIs bring appropriate experience in microbiome and asthma research in animal models and humans, and statistical and computational analysis.

The expertise provided by an international advisory board is a clear strength. Facilities were viewed as excellent for the proposed studies.

Sex and gender were taken into account

Overall, a clearly written proposal with well defined objectives.

Weaknesses:
The panel discussed the role of microbiota alterations, in extreme pre-term infants, during T cell ontogeny, in effecting immunological tolerance, and this was not discussed in the proposal. The mouse studies, therefore, may not yield appropriate data. However, the reviewers felt there was great potential in the study with the inclusion of these considerations

Additional details could have been provided on the applicability/translatability of the mouse model to human disease.
